# A high-efficiency discretized immersed boundary method for moving boundaries in incompressible flows

**DOI:** 10.1038/s41598-023-28878-5

**Published:** 2023-01-30

**Authors:** Dong Xu, Jianing Liu, Yunfeng Wu, Chunning Ji

**Affiliations:** 1 State Key Laboratory of Hydraulic Engineering Simulation and Safety, Tianjin University, Tianjin, 300072 China; 2 College of Water Conservancy & Hydropower Engineering, Hohai University, Nanjing, China

**Keywords:** Computational science, Hydrology, Particle physics

## Abstract

The Immersed Boundary Method (IBM) has an advantage in simulating fluid–structure interaction, owning to its simplicity, intuitiveness, and ease of handling complex object boundaries. The interpolation function plays a vital role in IBM and it is usually computationally intensive. For moving or deforming solids, the interpolation weights of all the immersed boundary points ought to be updated every time step, which takes quite a lot CPU time. Since the interpolation procedure within all uniform structured grids is highly repetitive and very similar, we propose a simple and generalized Discretized Immersed Boundary Method (DIBM), which significantly improves efficiency by discretizing the interpolation functions onto subgrid points within each control volume and reusing a predefined universal interpolation stencil. The accuracy and performance of DIBM are analyzed using both theoretical estimation and simulation tests. The results show speedup ratios of 30–40 or even higher using DIBM when compared with conventional IBM for typical moving boundary simulations like particle-laden flows, while the error is estimated to be under 1% and can be further decreased by using finer subgrid stencils. By balancing the performance and accuracy demands, DIBM provides an efficient alternative framework for handling moving boundaries in incompressible viscous flows.

## Introduction

Handling moving boundaries of complex shapes in flows has always been a challenging research topic for years, especially for the widely existing Fluid–structure Interaction (FSI) problems with moving boundaries^1–4^. Traditional FSI methods, like the arbitrary Lagrangian–Eulerian (ALE) method, work well with simple geometries but usually fail to deal with high grid distortions due to complex geometries. Fortunately, the emerging meshless FSI methods, especially the immersed boundary method (IBM), naturally avoids such defects and thus has a promising prospect^5,6^. IBM was first proposed by Peskin^7^ to simulate blood flow around the heart valves. The main idea of IBM is to simulate the interaction between fluid and structure by adding an extra body force to the Navier–Stokes (N–S) equations. Since the geometry of structure has been considered in the N–S equations using the body forces on immersed boundary points (IB points), there is no need to use the complex body-fitted mesh to describe the solid shapes^8,9^. Hence, a simple uniform Eulerian structured grid could be applied for spatial-discretizing the N–S equation, which makes boundary conditions easier to implement in computer programs.

According to the different ways of calculating the body force term, IBM is usually classified into the feedback-forcing approach and the direct-forcing approach^5,10^. The feedback-forcing approach solves the body force term according to the difference between calculated and expected displacement and velocity^11^. However, there is a limitation on the time step, and some empirical relaxation constants, which can only be decided case-to-case, are involved in determining the body force terms. The direct-forcing approach, proposed by Yusof^12^ and Fadlun et al.^13^, calculates the body force term directly from the momentum theorem. Usually, the velocity of IB points is an interpolation of the velocity of surrounding mesh grids. Using this direct-forcing IBM, the boundary conditions can be appropriately imposed without subjecting to the time step limitation by the solid rigidity^5^. The Navier–stokes solver itself is neither affected except for the additional body force terms, which means that the numerical methods, convergence, stability, and order of accuracy depends mainly on the original CFD solver without change. The direct-forcing IBM is widely used in complex FSI research due to its simplicity, intuitiveness, and ease of handling moving boundaries with complex geometries, such as blood flows containing red blood cells^14^, particle-laden flows^15^, flows with aquatic vegetations^16–19^ and also vortex-induced-vibration (VIV) phenomena^1,2^.

With the expending application of immersed boundary method in various research fields, more challenging issues about IBM have also emerged. One of them is the computational efficiency for imposing the boundary conditions via body force terms in the fluid solver. Although the explicit implementation of the boundary conditions under the framework of IBM is quite easy and straightforward, its computational costs cannot be overlooked. Such efficiency is even more crucial nowadays following the new trend of CFD development and applications. Firstly, the number of IB points is increasing rapidly with the growing scale of the numerical simulations. Take particle-laden multiphase flows for example, it is common to incorporate up to 10^5^–10^7^ IB points in just one simulation case^14,20^, whose computational cost is apparently non-negligible. Secondary, the most computationally expensive part of IBM, the calculation of the interpolation coefficients on each immersed boundary point, consumes an increasing computational time in nowadays’ FSI simulations^21–23^. For the flow simulations with moving boundaries or deforming solids, the interpolation coefficients of all the IB points ought to be recomputed at each time step. This process further increases the computational cost of IBM functions. Thirdly, more and more complicated interpolation functions are proposed by many researchers for different purposes, including the piecewise, quadratic, Cosine smooth and so on^5,24^. These new interpolation schemes successfully improve computational accuracy in various contexts via advanced high order approximations^6,25^. Nevertheless, such advanced schemes are usually computationally expensive and difficult to implement due to its high complexity in algorithm. It is also unfavorable for parallelized computing using modern graphical units (GPUs), which is more suitable for handling massive data rather than complex algorithms^26,27^.

This study introduces the discretized immersed boundary method (DIBM) with the purpose of improving the computational efficiency and simplifying the implementation of the IBM technique. The main idea of DIBM is to further discretize the Eulerian grid cells containing IB points into finer subgrid points and then precompute and store their interpolation coefficients during initialization. Spandan et al.^28^ proposed a similar concept with Lagrangian maker and sub-Eulerian nodes, which are used in a fast moving least squares approximation with adaptive Lagrangian mesh refinement for large scale immersed boundary simulations. However, interpolation of the coefficients of each immersed boundary point remains a necessary part in Spandan et al.^28^, which is supposed to be implemented using the tri-linear interpolation or the Shepard’s interpolation. In contrast, the DIBM method proposed here does not compute any interpolation coefficients at each time step, but takes values on subgrid directly from prestored discretized stencil. During the imposition of IBM boundary, the original IB points will be mapped onto the subgrid points, namely discretized immersed boundary points or DIB points, use the nearest neighbor principle. Then the interpolation coefficients can be extracted directly from the precomputed storage, rather than repeating the real interpolation process, which is computationally expensive. In another word, the interpolation process of IBM functions is stenciled using finer subgrid points inside a standard unit of control volume. For uniform structured Cartesian grids, which are the mostly adopted by IBM, such stencils exist and can be easily generated by normalizing the grid cell into a unit cell and then divide it into finer subgrids using smaller spacings. In this way, the computational cost of the interpolation of IB points can be mostly saved because the interpolation functions, either simple first-order ones or more complex higher-order ones, need only be called once for all during initialization. During implemented, the interpolation coefficients can also be precomputed using other specially designed codes or other advanced mathematical tools, and loaded into the CFD solver via data files during initialization. Such treatment also greatly simplified the implementation of the interpolation function of IBM, especially for the parallelized computing on data-efficient modern GPUs. This also facilitates the implantation and share of future newly developed interpolation functions or IBM algorithms using a generalized procedure with stenciled coefficients. However, such an alternative treatment of IBM implementation raises two new questions: the first is that how much accuracy will be lost during the mapping of the original IB points onto nearest subgrid points, namely DIB points? The second is how much efficiency improvement will be achieved using the new method—the DIBM when compared against conventional IBM?

The answer to the above two questions will be investigated in this study. As for the first question, the numerical error introduced by the stenciled treatment of the interpolation coefficients will be evaluated using both theoretical estimations and numerical tests. The second question will be discussed using test runs with typical particle-laden flows directly. This paper is organized as follows: the algorithm of DIBM will be discussed in “Numerical methods” section. In “Analysis on accuracy and efficiency” section, the accuracy and performance of DIBM will be analyzed with comparison to IBM with some idealized assumptions, and the real behavior on accuracy and performance with be tested using a series of validation case presented in “Case studies and discussions” section. The conclusions are summarized in “Conclusions” section.

## Numerical methods

### Governing equations

The DIBM method is compatible with all CFD solvers under the framework of Cartesian structured grids. For simplification, its development and application will be presented and implemented using our in-house code for incompressible viscous flows -CgLES^5^. The governing equations for incompressible viscous flows, known as the N–S equation, are:

1 $$\frac{{{\mathbf{du}}}}{{{\mathbf{d}}t}} = - \frac{1}{\rho }\nabla p + {\mathbf{f}} + \nu \nabla^{2} {\mathbf{u}},$$

and

2 $$\nabla \cdot {\mathbf{u}} = 0,$$

where, **u** is the velocity, *p* is the pressure, **f** is the body force term, and *t* is time. *ρ* and *ν* are the density and kinematic viscosity of the liquid, respectively. And $\nabla$ denotes the nabla operator. Notice that $\frac{{{\mathbf{du}}}}{{{\mathbf{d}}t}} = \frac{{\partial {\mathbf{u}}}}{\partial t} + \left( {{\mathbf{u}} \cdot \nabla } \right){\mathbf{u}}$, substitute it into Eq. (1), we have

3 $$\frac{{\partial {\mathbf{u}}}}{\partial t} = - \frac{1}{\rho }\nabla p + {\mathbf{f}} + {\mathbf{h}},$$

where ${\mathbf{h}} = - \left( {{\mathbf{u}} \cdot \nabla } \right){\mathbf{u}} + \nu \nabla^{2} {\mathbf{u}}$. Note that both the body force term **f** and the convective and diffusive terms **h** depend on the velocity **u**. The projection method is used to decouple the velocity and the pressure terms. The equations are solved on staggered structured grid using second order finite difference discretization. The Adams–Bashforth scheme is used for temporal discretization, which has also second order accuracy. The pressure Poisson equation is solved using the Conjugated Gradient Method with multigrid accelerations. More details on the numerical method can be found in our previous works^5^.

### Immersed boundary method

The framework of direct-forcing immersed boundary method^5^ is adopted. To solve the governing equations, suppose ${\mathbf{u}}^{n}$ as the velocity at the current time step and (${\mathbf{u}}^{n - 1}$,$p^{n - 1}$) as the velocity and pressure at the last time step, which are known. Thus the pressure at the current time step $p^{n}$ and the velocity at the next step ${\mathbf{u}}^{n + 1}$ need to be solved. The left-hand side of Eq. (3) means the change of velocity during time iteration, and it can be replaced by ${\mathbf{u}}^{n + 1}$ and ${\mathbf{u}}^{n}$:

4 $$\frac{{\partial {\mathbf{u}}}}{\partial t} = \frac{{{\mathbf{u}}^{n + 1} - {\mathbf{u}}^{n} }}{\delta t},$$

where *δt* is the time step. $p^{{n + \frac{1}{2}}} {, }{\mathbf{f}}^{{n + \frac{1}{2}}} {, }{\mathbf{h}}^{{n + \frac{1}{2}}}$ denote the pressure term, the body force term, and the convective and diffusive terms, respectively, which contribute to the change of velocity during iteration. Using the Adams–Bashforth time discretization, the momentum equations read:

5 $${\mathbf{u}}^{n + 1} = {\mathbf{u}}^{n} + \delta t\left( { - \frac{1}{\rho }\left( {\frac{3}{2}p^{n} - \frac{1}{2}p^{n - 1} } \right) + \frac{3}{2}{\mathbf{h}}^{n} - \frac{1}{2}{\mathbf{h}}^{n - 1} } \right) + \delta t{\mathbf{f}}^{{n + \frac{1}{2}}} ,$$

One of the essential parts of IBM is to determine the body force term of Eq. (5). Denote $I\left( \phi \right)$ and $D\left( \Phi \right)$ as the interpolation and distribution function, respectively. Here, the lower-case $\phi$ is the variable at grid points, such as ${\mathbf{u}}{, }p{, }{\mathbf{f}}$, etc., while the upper-case $\Phi$ is the variable at IB points, such as ${\mathbf{U}}{, }P{, }{\mathbf{F}}$, etc. And the boundary condition in IBM is:

6 $$I\left( {{\mathbf{u}}^{n + 1} } \right) = {\mathbf{U}}^{n + 1} ,$$

where $I\left( {{\mathbf{u}}^{n + 1} } \right)$ is the interpolated velocity at IB points and ${\mathbf{U}}^{n + 1}$ is the desired velocity at IB points. Substitute Eq. (5) into Eq. (6) gives

7 $${\mathbf{U}}^{n + 1} = I\left( {{\mathbf{u}}^{n + 1} } \right) = I\left( {{\mathbf{u}}^{n} + \delta t\left( { - \frac{1}{\rho }\left( {\frac{3}{2}p^{n} - \frac{1}{2}p^{n - 1} } \right) + \frac{3}{2}{\mathbf{h}}^{n} - \frac{1}{2}{\mathbf{h}}^{n - 1} } \right)} \right) + I\left( {\delta t{\mathbf{f}}^{{n + \frac{1}{2}}} } \right),$$

The equation above can be rewritten as:

8 $$\delta t{\mathbf{F}}^{{n + \frac{1}{2}}} = I\left( {\delta t{\mathbf{f}}^{{n + \frac{1}{2}}} } \right) = {\mathbf{U}}^{n + 1} - I\left( {{\mathbf{u}}^{n} + \delta t\left( { - \frac{1}{\rho }\left( {\frac{3}{2}p^{n} - \frac{1}{2}p^{n - 1} } \right) + \frac{3}{2}h^{n} - \frac{1}{2}{\mathbf{h}}^{n - 1} } \right)} \right),$$

where ${\mathbf{F}}^{{n + \frac{1}{2}}}$ is the flow-induced body force imposed on the IB points. Then, distribute the body force into the Cartesian grids:

9 $$D\left( {\delta t{\mathbf{F}}^{{n + \frac{1}{2}}} } \right) = D\left( {I\left( {\delta t{\mathbf{f}}^{{n + \frac{1}{2}}} } \right)} \right) = D\left( {{\mathbf{U}}^{n + 1} - I\left( {{\mathbf{u}}^{n} + \delta t\left( { - \frac{1}{\rho }\left( {\frac{3}{2}p^{n} - \frac{1}{2}p^{n - 1} } \right) + \frac{3}{2}{\mathbf{h}}^{n} - \frac{1}{2}{\mathbf{h}}^{n - 1} } \right)} \right)} \right),$$

Finally, the body force term is defined as:

10 $$\delta t{\mathbf{f}}^{{n + \frac{1}{2}}} = D\left( {{\mathbf{U}}^{n + 1} - I\left( {{\mathbf{u}}^{n} + \delta t\left( { - \frac{1}{\rho }\left( {\frac{3}{2}p^{n} - \frac{1}{2}p^{n - 1} } \right) + \frac{3}{2}{\mathbf{h}}^{n} - \frac{1}{2}{\mathbf{h}}^{n - 1} } \right)} \right)} \right),$$

The above implementation of IBM has been incorporated into our in-house code -CgLES and well validated using a series of simulation cases^5^. It has been successfully applied in different fields including hydraulic engineering, ocean engineering and biomechanical engineering^1,2,14^.

### The interpolation function

In the original immersed boundary method, the interpolation function is one of the most important kernel of computation. A discretized Dirac delta function^7^ $\phi \left( r \right)$ is widely used for interpolation, where *r* is the distance between an immersed point and the surrounding fluid grid point normalized by the grid size *h*. It satisfies

11 $$\left\{ {\begin{array}{*{20}l} {\sum\limits_{jeven} {\phi \left( {r - j} \right) = \sum\limits_{jodd} {\phi \left( {r - j} \right) = 0.5} } } \hfill & {\left| r \right| < 2} \hfill \\ {\sum\limits_{j} {\left( {r - j} \right)\phi \left( {r - j} \right) = 0} } \hfill & {\left| r \right| < 2} \hfill \\ {\phi \left( r \right) = 0} \hfill & {\left| r \right| \ge 2} \hfill \\ \end{array} } \right.,$$

For the Quadratic interpolation function and Cosine-smooth interpolation function for example, the interpolation coefficient is calculated using,

12 $$\phi \left( r \right) = \left\{ {\begin{array}{*{20}l} {\frac{1}{8}\left( {3 - 2\left| r \right| + \sqrt {1 + 4\left| r \right| - 4r^{2} } } \right),} & {\left| r \right| \le 1} \\ {\frac{1}{8}\left( {5 - 2\left| r \right| - \sqrt { - 7 + 12\left| r \right| - 4r^{2} } } \right),} & {1 < \left| r \right| < 2} \\ {0,} & {\left| r \right| \ge 2} \\ \end{array} } \right.,$$

13 $$\phi \left( r \right) = \left\{ {\begin{array}{*{20}l} {\frac{1}{4}\left( {1 + \cos \left( {\frac{\pi \left| r \right|}{2}} \right)} \right)} \hfill & {\left| r \right| < 2} \hfill \\ 0 \hfill & {\left| r \right| \ge 2} \hfill \\ \end{array} } \right.,$$

Computation of the interpolation coefficient on immersed boundary points using the above interpolation functions involves complex algebraic operations including exponential and trigonometric functions, which are usually computationally heavy tasks for computers. A stenciled treatment of the interpolation coefficients based on a normalized unit grid cell is possible to speedup this process.

### Discretized immersed boundary method

The discretized immersed boundary method (DIBM) improves the computational efficiency by avoiding the repetitive calculation of the interpolation coefficients of IB points in IBM when dealing with moving boundaries. The main idea of DIBM is to discretize the mesh cell containing IB points, namely the basic grid, into finer subgrids and then use the nearest subgrid points, called DIB points, to replace the actual IB points. Thus, calculation of the IB points’ interpolation coefficients can be replaced by matching the nearest discretized subgrids and getting the corresponding coefficients from the prestored database. In this way, DIBM greatly reduces the computational costs of moving boundary treatment. Particularly for moving objects with a large number of IB points and complicated interpolation functions, the time consumed by coefficient interpolation could be dramatically reduced. Note that the direct-forcing immersed boundary method adds body force terms into the momentum equations in an explicit way and such treatment does not affect the Navier–stokes solver in practice. Therefore, the stability and the accuracy of the solver is not affected in general.

Taking a two-dimensional case for example, the steps of DIBM implementation are:

***Prepare the interpolation stencil for subgrid DIB points*** *:*

**Figure 1:**
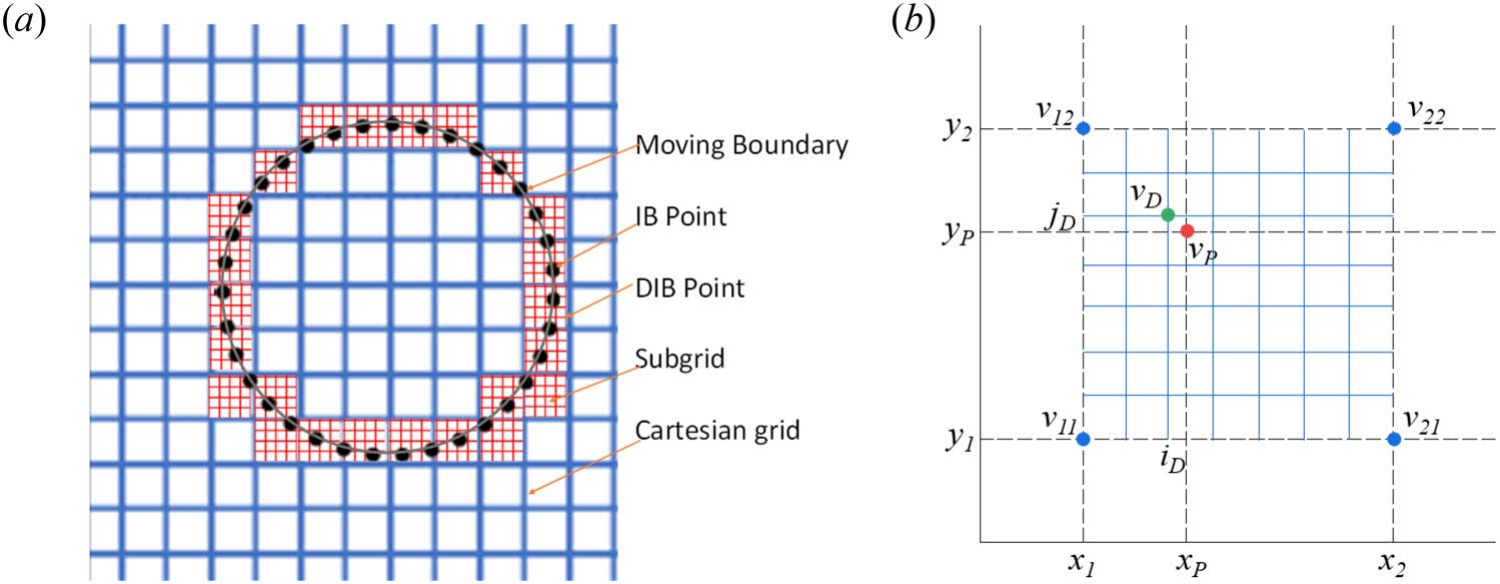
Schematic of the interpolation using discretized immersed boundary method (**a**) overall grid (**b**) within one grid cell.

(a) Given the subgrid point number $N$ in each individual direction, the coordinates of each subgrid are

14 $$P_{subgrid} = \left( {x_{1} + \frac{i}{N}d,y_{1} + \frac{j}{N}d} \right){, }i = 0,1,2,...N - 1{; }j = 0,1,2,...N - 1,$$

where $\left( {x_{1} ,y_{1} } \right)$ is the coordinate of the lower-left grid of the mesh cell and $d$ is the mesh size, as shown in Fig. 1.

(b) Calculate the interpolation coefficients $C$ for each discretized grid according to the interpolation function. For a bilinear piecewise interpolation under a staggered grid configuration, there are 10 coefficients for each subgrid point: $i$, $j$, $Cx_{{\left( {x_{1} ,y_{1} } \right)}}$, $Cx_{{\left( {x_{1} ,y_{2} } \right)}}$, $Cx_{{\left( {x_{2} ,y_{1} } \right)}}$, $Cx_{{\left( {x_{2} ,y_{2} } \right)}}$, $Cy_{{\left( {x_{1} ,y_{1} } \right)}}$, $Cy_{{\left( {x_{1} ,y_{2} } \right)}}$, $Cy_{{\left( {x_{2} ,y_{1} } \right)}}$, and $Cy_{{\left( {x_{2} ,y_{2} } \right)}}$, where $i$ and $j$ are the index of the current discretized grid in the *x*- and *y*-direction, respectively; $Cx_{{\left( {x,y} \right)}}$ and $Cy_{{\left( {x,y} \right)}}$ are the interpolation coefficients for the fluid velocity at basic grid in the *x*- and *y*-directions, respectively.

(c) Save the precomputed interpolation coefficients into a global array or a data file. Note that for given subgrid resolution and selected interpolation function, the coefficient array need only be built once targeting at a normalized unit-size control volume.

***Run the CFD solver***

(a) Read the Cartesian coordinates of the IB points from the data file into an array $P$.

(b) Read the interpolation coefficients from the precomputed and saved coefficient data file into an array $C$.

(c) Set the subgrid point number *N* and grid size *d*.

(d) Initialize the flow fields, including velocity components, pressure, and so on.

***Begin loop***

(i) Calculate the intermedia velocity ${\mathbf{u}}^{*}$;
(ii) For each IB point, calculate the *x*- and *y*-direction global index of the control volume containing this IB points.
(iii) Map the IB point inside the control volume onto a subgrid point, namely the DIB point. Extract the precomputed interpolation coefficients corresponding to this DIB point in the global array *C*.
(iv) Calculate the interpolation velocity ${\mathbf{U}}^{n + 1}$ on the DIB points according to the interpolation coefficients.
(v) Calculate the body force terms corresponding to immersed boundary point ${\mathbf{f}}^{{n + \frac{1}{2}}}$ ;
(vi) Update the intermedia velocity ${\mathbf{u}}^{*}$ using the above body force terms;
(vii) Calculate the current pressure $p^{n}$ using pressure Poisson equation solver;
(viii) Calculate the velocity at the next time step ${\mathbf{u}}^{n + 1}$ by pressure correction;
(ix) For moving boundaries, reposition the IB points and repeat the previous steps from (i).

***End loop***

(e) Output necessary data into data files.

***End of the solver***

## Analysis on accuracy and efficiency

### Interpolation error

The fundamental idea of DIBM is replacing the interpolation coefficients for the physical immersed boundary points by the precomputed stenciled coefficients for the nearest subgrid points, which can be viewed as virtual points. This treatment obviously introduces errors for the representation of the actual solid boundaries on a subgrid level. Just like the staircase approximation of wall boundaries in structured Catesian grids^29^, the error decreases when the subgrid resolution increases. To quantify this error theoretically, let take a 2D bilinear interpolation function for example. If the control volume is further discretized into *N*x*N* subgrid points, the interpolated velocity at each subgrid point using the bilinear interpolation function is:

15 $$\begin{gathered} v_{p} = \left( {1 - k_{x} } \right)\left( {1 - k_{y} } \right)v_{11} + k_{x} \left( {1 - k_{y} } \right)v_{21} + \left( {1 - k_{x} } \right)k_{y} v_{21} + k_{x} k_{y} v_{22} {,} \hfill \\ k_{x} = \frac{{x_{p} - x_{1} }}{{x_{2} - x_{1} }}{, }k_{y} = \frac{{y_{p} - y_{1} }}{{y_{2} - y_{1} }} \hfill \\ \end{gathered}$$

where $v_{ij}$ is the flow velocity at grid points $\left( {x_{i} ,y_{j} } \right){, }i = 1,2{, }j = 1,2$; and $v_{p}$ is the interpolation velocity at IB points $\left( {x_{p} ,y_{p} } \right)$, $x_{1} < x_{p} < x_{2}$, $y_{1} < y_{p} < y_{2}$, $x_{2} - x_{1} = y_{2} - y_{1} = d$. Divide the mesh into $N \times N$ discretized cells. Suppose that an immersed boundary point be located inside a control volume, as shown in Fig. 1, its nearest neighboring subgrid point is $P\left( {{\text{DIB}}} \right) = \left( {x_{1} + \frac{{i_{D} }}{N}d,y_{1} + \frac{{j_{D} }}{N}d} \right)$.

In the worst case, the coordinate of the real IB point would be half of subgrid size away from corresponding DIB point, namely $P\left( {{\text{IB}}} \right) = \left( {\frac{{i_{D} \pm 1/2}}{N}d,\frac{{j_{D} + 1/2}}{N}d} \right)$. Substitute the coordinates of $P\left( {{\text{IB}}} \right)$ and $P\left( {{\text{DIB}}} \right)$ into Eq. (15) gives:

16 $$\begin{gathered} v_{p} = \left( {1 - \frac{{i_{D} \pm 1/2}}{N}} \right)\left( {1 - \frac{{j_{D} \pm 1/2}}{N}} \right)v_{11} + \frac{{i_{D} \pm 1/2}}{N}\left( {1 - \frac{{j_{D} \pm 1/2}}{N}} \right)v_{21} \hfill \\\quad\quad + \left( {1 - \frac{{i_{D} \pm 1/2}}{N}} \right)\frac{{j_{D} \pm 1/2}}{N}v_{21} + \frac{{i_{D} \pm 1/2}}{N}\frac{{j_{D} \pm 1/2}}{N}v_{22} , \hfill \\ \end{gathered}$$

and

17 $$v_{D} = \left( {1 - \frac{{i_{D} }}{N}} \right)\left( {1 - \frac{{j_{D} }}{N}} \right)v_{11} + \frac{{i_{D} }}{N}\left( {1 - \frac{{j_{D} }}{N}} \right)v_{21} + \left( {1 - \frac{{i_{D} }}{N}} \right)\frac{{j_{D} }}{N}v_{21} + \frac{{i_{D} }}{N}\frac{{j_{D} }}{N}v_{22} ,$$

During computation of the interpolation coefficients, it is the differences among the neighbor values $v_{11} {, }v_{21} {, }v_{12} {, }v_{22}$ lead to the interpolation error, namely $v_{p}$—$v_{D}$, rather than their uniform average values. Using the standard deviation, namely ${\text{std}}\left( {v_{11} {, }v_{21} {, }v_{12} {, }v_{22} } \right)$, of the flow velocity of surrounding basic grids as the basis for error normalization, the relative difference between the interpolated value at IB point—$v_{p}$ and at the corresponding DIB point—$v_{D}$, *v*_*error*_, can be estimated as:

18 $$v_{error} = \frac{{\left| {v_{p} - v_{D} } \right|}}{{std\left( {v_{11} ,v_{12} ,v_{21} ,v_{22} } \right)}},$$

where the standard deviation is defined as $std\left( {v_{11} ,v_{12} ,v_{21} ,v_{22} } \right) = N^{2} \sqrt {\sum v_{ij}^{2} } /8$ and

19 $$\begin{gathered} \sum v_{ij}^{2} = \left( { - 3v_{11} + v_{12} + v_{21} + v_{22} } \right)^{2} + \left( {v_{11} - 3v_{12} + v_{21} + v_{22} } \right)^{2} \hfill \\ + \left( {v_{11} + v_{12} - 3v_{21} + v_{22} } \right)^{2} + \left( {v_{11} + v_{12} + v_{21} - 3v_{22} } \right)^{2} , \hfill \\ \end{gathered}$$

The value of dimensional interpolation error can be estimated as

20 $$\begin{aligned} v_{p} - v_{D} = & \,v_{11} \left( {i_{D} - N \pm \frac{1}{2}} \right)\left( {j_{D} - N \pm \frac{1}{2}} \right) - v_{12} \left( {j_{D} \pm \frac{1}{2}} \right)\left( {i_{D} - N \pm \frac{1}{2}} \right)\\ &- v_{21} \left( {i_{D} \pm \frac{1}{2}} \right)\left( {j_{D} - N \pm \frac{1}{2}} \right) + v_{22} \left( {i_{D} \pm \frac{1}{2}} \right)\left( {j_{D} \pm \frac{1}{2}} \right) - i_{D} j_{D} v_{22} \\& + i_{D} v_{21} \left( {j_{D} - N} \right) + j_{D} v_{12} \left( {i_{D} - N} \right) - v_{11} \left( {i_{D} - N} \right)\left( {j_{D} - N} \right), \\ \end{aligned}$$

**Figure 2:**
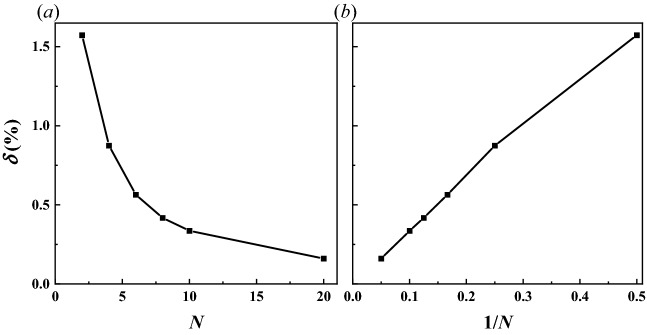
Mean normalized interpolation error of DIBM against IBM (**a**) error versus subgrid number (**b**) error versus subgrid spacing.

The numerator of the above formula scales as *O*(1) while the denominator scales as *O*(*N*^−2^). Hence, the normalized interpolation error scales as *O*(*N*^−2^). As long as the subgrid resolution is sufficiently high, namely a *N* value large enough, the interpolation error caused by the stenciled approximation in DIBM will be low and negligible. Considering that the immersed boundary points of solid boundaries are usually a set of scatter points randomly distributed in the computational domain, therefore the deviation of the position of different IB points from their stenciled DIB counterparts are also randomly different. This leads to a randomly distributed interpolation error, rather than the maximum error estimated using Eq. (15). A more reasonable estimation of the overall error level could be the mean value of all the points. To get such statistical evaluations, a test case was configured with totally 10,000 IB points randomly distributed in a cubic computational domain with edge size of 1. For stenciled interpolation using different subgrid resolutions, the errors in interpolation coefficients of all the IB points were measured and their mean value −*δ* was calculated, see Fig. 2. The mean normalized interpolation error *δ* decreases rapidly as the subgrid resolution improves, demonstrating an approximately linear relationship with subgrid space 1/*N*. For a subgrid point number *N* = 5, the mean normalized interpolation error is lower than 1%; the error further decreases to around 0.3% for a subgrid point number *N* = 10, which is sufficient for most numerical simulation cases with moving boundaries.

### Efficiency improvement

The efficiency improvement of the proposed DIBM over the conventional IBM is apparent. Because DIBM precomputes all the interpolation coefficients and reuses them in the following iterations, all the computational cost by the interpolation functions of the conventional IBM is then saved. Of course, there shall be an overhead computational cost for looking up the index of the precomputed interpolation coefficients and read them from a preset global array. However, such extra computations are usually very cheap under the framework of uniform structured basic grid and subgrid. The computational cost for the interpolations is composed of several subroutine calls, which can be further breakdown into basic algebraic operations including add, subtraction, multiplication, and basic functions like square root and cosine and so on. For different interpolation functions, like linear, quadratic and cosine smooth etc., the operation count statistics for basic operation and function calls are counted and presented in Table 1. For a simple linear interpolation in three-dimensional simulations, namely *m* = 3, there will be 7 × 3 = 21 add/sub operations and 3 × 3 = 9 multiplication operations; for a more complex Cosine smooth interpolation, there will be 38 × 3 = 114 add/sub operations, 27 × 3 = 81 multiplication operations and 16 × 3 = 48 cosine function calls. However, for a DIBM implementation, only 8 add/sub operations and 29 multiplication operations are required by the function for extracting interpolation coefficients from the precomputed array. What more, the basic arithmetic operation used by the DIBM method is much cheaper than the advanced function calls like square root or cosine used by more advanced interpolation functions. The actual performance improvement will be tested using a series of simulation runs in the following sections.

**Table 1:** Arithmetic operation count required by different methods.

Method	Interpolation function	Add/Sub	Mul	Div	Sqrt	Cos	Total
IBM	Linear	7 m	3 m	–	–	–	10 m
	Quadratic (Peskin, 2002)	22 m	17 m	–	2 m	–	41 m
	Cosine_smooth (Yang et al., 2009)	38 m	27 m	–	–	16 m	81 m
DIBM	*	3 m^−1^	4 m^−3^	–	–	–	7 m^−4^

The symbol “*” means application for any kind of interpolation function. *m* refers to the unit count for a basic CPU operation in a one-dimensional problem, which equals 2 for two-dimensional cases and 3 for three-dimensional cases.

## Case studies and discussions

### Flow around a stationary and a moving cylinder

The overall performance and accuracy of the proposed DIBM are validated using different simulation test runs. The accuracy of DIBM is verified using a flow around a fixed cylinder. As for the efficiency, DIBM does not show apparent improvement for stationary solid boundaries because the interpolation coefficients need not to be updated during time iterations. Therefore, flow with a moving cylinder is adopted for the test purpose. All the simulation results using DIBM will be compared to that of IBM, and the effects of the number of IB points and the complexity of the interpolation function on the simulation efficiency will also be discussed. For simplification, only laminar flows will be simulated at current stage.

**Figure 3:**
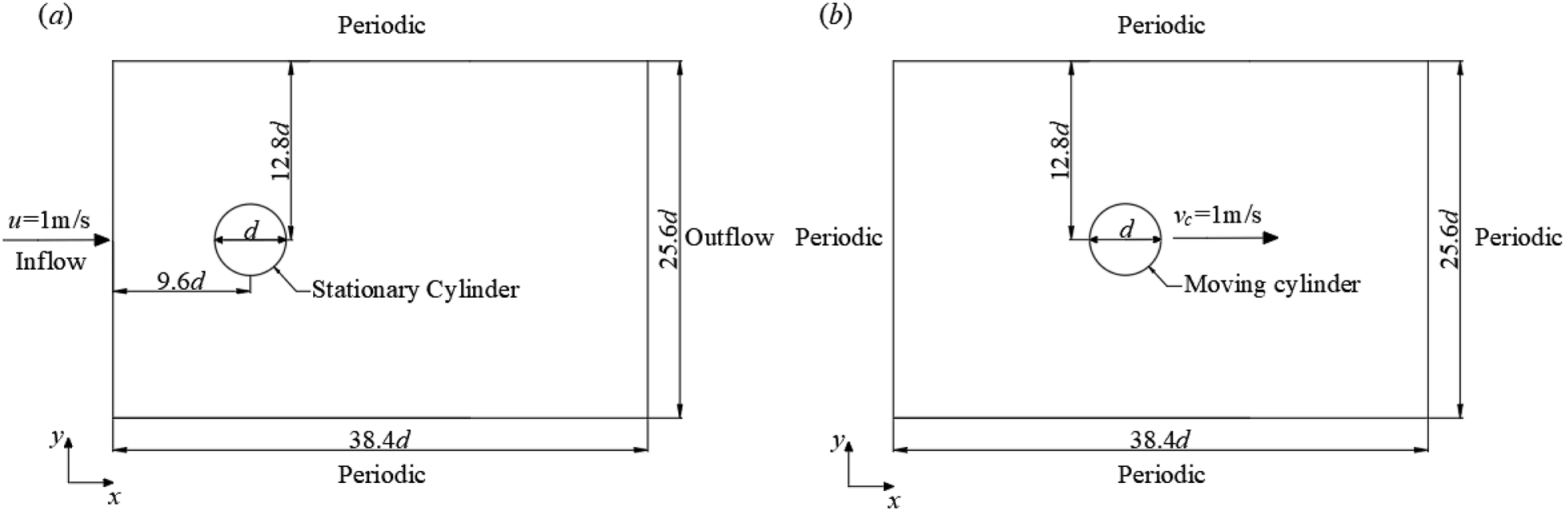
Schematics for simulation setup for flow around a cylinder (**a**) stationary cylinder (**b**) moving cylinder.

For the flow around a cylinder, the simulation case is configured following Ji et al.^5^. The length—*L* and width—*W* of the computational domain are $38.4d$ and $25.6d$, respectively, with $d = 0.625{\text{cm}}$ being the diameter of the cylinder. The center of the cylinder is located at $\left( {9.6d,12.8d} \right)$ as shown in Fig. 3. The boundary conditions in spanwise direction are periodic, while in streamwise, the left boundary is the inflow boundary, and the right one is outflow boundary. The size of the time step and kinematic viscosity coefficient is chosen according to the CFL number and the Reynolds number, respectively. A relatively low Reynolds number, Re = 100 is adopted in the simulations, which means the flow is laminar. Unless otherwise specified, all below simulations adopted such configurations.

For the case with a stationary cylinder, the inflow velocity is $u = 1{\text{cm/s}}$, and the basic grid size is fine enough to ensure that there are 40 grids within the cylinder’s diameter. For the moving cylinder, the center of the cylinder is first located at $\left( {9.6d,12.8d} \right)$ and then moves from left to right with a velocity of $v_{c} = 1{\text{cm/s}}$. More details are listed in Table 2.

**Table 2:** Simulation cases for flow around stationary or moving cylinder using both DIBM and IBM.

Case	Method	Subgrid point of DIBM ($N$)	Number of IB points ($N_{{{\text{IBP}}}}$)	Interpolation function
Sc.1	IBM	–	2000	Linear
Sc.2	DIBM	2		
Sc.3	DIBM	4		
Sc.4	DIBM	6		
Sc.5	DIBM	8		
Sc.6	DIBM	10		
Sc.7	DIBM	20		
Mc.1	IBM	^−^	5 × 10^4^, 1 × 10^5^, 2 × 10^5^, 4 × 10^5^, 8 × 10^5^	Linear
Mc.2	IBM	^−^		Quadratic
Mc.3	IBM	^−^		Cosine_smooth
Mc.4	DIBM	10		Linear
Mc.5	DIBM	10		Quadratic

Case Sc refers to the case with a stationary cylinder; Case Mc refers to the case with a moving cylinder.

**Figure 4:**
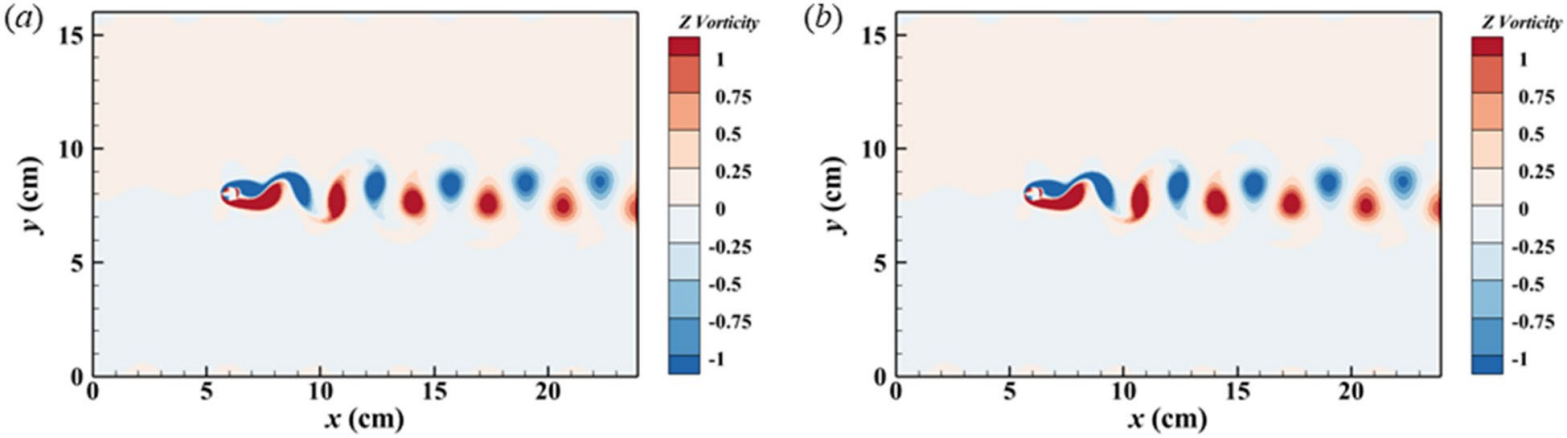
Contour of Z-vorticity of flow around a stationary cylinder (Case Sc.6) (**a**) IBM (**b**) DIBM.

Simulations using both the IBM methods and the DIBM methods well reproduced the Karman Vortex Streets behind the stationary cylinder and the flow fields are basically the same in the contour plots shown in Fig. 4. Quantitative verifications of simulation results are also provided, see Table 3. For the flow around a stationary cylinder, the drag force coefficient $C_{D}$, lift force coefficient $C_{L}$, and the Strouhal number $St$ are computed using Eq. (21)–(23). The simulation results by DIBM are compared with those using IBM and with other published data, as shown in Table 3. The results of case Sc.1 using IBM agree well with Ji et al.^5^, with a relative error of 2.87% in $C_{D}$, 2.09% in $C_{L}$, and 0.30% in $St$. As the subgrid point number grows from $N = 2$ to $N = 20$, the relative error between IBM and DIBM in $C_{D}$ decreases from 4.99 to 1.05%, while the relative error in $C_{L}$ decreases from 6.43 to 2.05%. The relative error of $St$ is as low as 0.60%.

21 $$C_{D} = \frac{{2F_{D} }}{{\rho u^{2} d}}$$

22 $$C_{L} = \frac{{2F_{L} }}{{\rho u^{2} d}}$$

23 $$St = \frac{fd}{u}$$

where the $F_{D}$ and $F_{L}$ is the drag force and lift force of the cylinder, respectively; and $\rho$ is the density of the fluid, $u$ is the inflow velocity, and $f$ is the frequency of vortex shedding.

**Table 3:** Comparison of drag force and lift force coefficient, and Strouhal number of flows around a cylinder at Re = 100.

Source	Case	$C_{D}$	$\delta_{{C_{D} }}$	$C_{L}$	$\delta_{{C_{L} }}$	$St$	$\delta_{St}$
Present simulations	Sc.1	1.422	−2.87%	0.342	2.09%	0.168	0.30%
	Sc.2	1.493	4.99%	0.335	−2.05%	0.167	−0.60%
	Sc.3	1.456	2.39%	0.326	−4.68%	0.167	−0.60%
	Sc.4	1.462	2.81%	0.326	−4.68%	0.167	−0.60%
	Sc.5	1.441	1.34%	0.321	−6.14%	0.167	−0.60%
	Sc.6	1.437	1.05%	0.321	−6.14%	0.167	−0.60%
	Sc.7	1.439	1.20%	0.320	−6.43%	0.167	−0.60%
Literatures	Uhlmann^30^	1.453	–	0.339	–	0.169	–
	Tseng et al.^31^	1.420	–	0.290	–	0.164	–
	Ji et al.^5^	1.464	–	0.335	–	0.168	–

**Figure 5:**
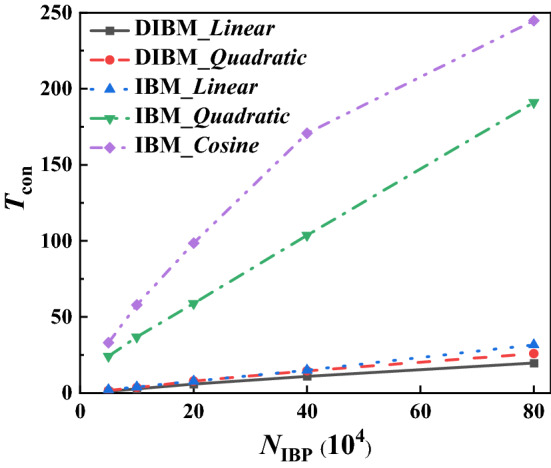
CPU time consumption per step by DIBM and IBM.

In order to find out the CPU time consumption following the increasing of IB points, test runs with randomly seeded IB points ranging from 50, 000 to 800, 000 are performed using other configurations the same as the above Sc.1. The performances of both DIBM and IBM using different interpolation functions and different numbers of IB points are shown in Fig. 5. The CPU time is profiled during the entire simulation and the part for immersed boundary treatment is specially extracted, which is composed of the subroutines for interpolation looking up, namely sub-step (ii) and (iii) in step (d) of DIBM implementation; For conventional IBM, it refers to the computational time for coefficients interpolation. Such CPU time consumption is summed up to 1000 times steps and their average values per time step are then calculated. Note that all the data in Fig. 5 has been normalized with the results of Case Mc.4 ($N_{{{\text{IBP}}}} = 5 \times 10^{4}$), which requires the minimum computations. The relative CPU time consumption per step $T_{{{\text{con}}}}$ is roughly proportional to the number of the IB points using different interpolation functions for IBM. For all the cases, the computational cost of DIBM is much lower than its IBM counterpart. This is especially true for high order interpolation functions like quadratic, for which DIBM is about 34 times faster than IBM. For simple linear interpolation, the acceleration rate is not that high, which is related to the extra memory accessing time cost by DIBM. Another fascinating fact is shown in Table 4, which demonstrates that as the number of IB points increases 16 times, the time consumption of Case Mc.4 grows 16.03 and 19.69 for the DIBM cases, which are the closest to a linear growth when compared with that of other cases using IBM. This implies that the proposed DIBM have scalability higher than the conventional IBM when the computational scale increases. The CPU time required for other parts of the solver, including the momentum equations, the pressure Poisson equation, and the boundary treatment, is over 5.75 times of the IBM subroutines (case Mc.3) in all the tests, which implies that the immersed boundary treatment always requires a relatively small proportion of the CPU time of the entire solver. Note that this proportion is highly dependent on the pressure solver adopted. Our comparison here focuses more on the efficiency improvement of DIBM against IBM. What more, with the increasing of the IB points, the proportion of CPU time by IBM will further increase.

**Table 4:** Comparison of the growth ratio of CPU time consumption against computational scale.

Number of IB points	Mc.1(IBM)	Mc.2(IBM)	Mc.3(IBM)	Mc.4(DIBM)	Mc.5(DIBM)
	IBM_linear	Ibm_quadratic	Ibm_cosine	DIBM_linear	DIBM_quadratic
5 × 10^4^	2.20	24.15	33.02	1.00	1.62
1 × 10^5^	3.76	30.19	41.94	1.98	3.29
2 × 105	7.10	47.82	62.40	4.93	6.79
4 × 10^5^	15.58	98.29	130.76	9.91	13.15
8 × 10^5^	31.72	190.97	244.80	19.69	25.90
Growth ratio*	14.40	7.91	7.41	19.69	16.03

*Note: Growth ratio is estimated using 8 × 10^5^ IB points with reference to the case Mc_4 with the minimum IB points.

### Settling of a single and multiple particles

**Figure 6:**
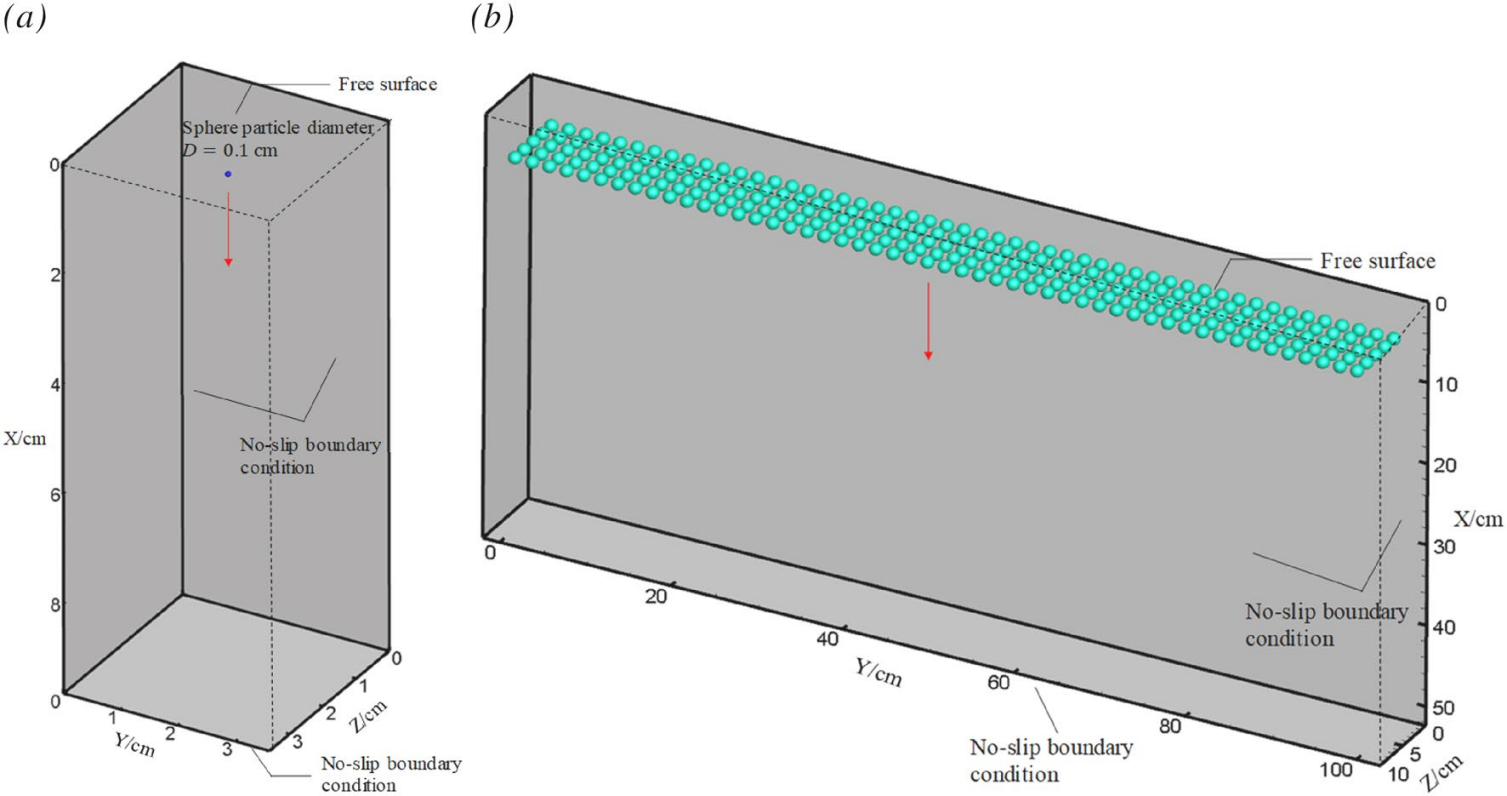
Schematic of the computational domain for particle setting (**a**) single particle (**b**) multiple particles.

To verify the results of simulations for fluid–solid interactions, a simple case for particle settling in still water is conducted using both IBM and DIBM. Both of the simulations are particle shape-resolved and the results are compared against the experiment conducted by Mordant et al.^32^. The simulation domain is sized as 36D×36D×96D, where D = 0.1 cm is the diameter of the spherical particle. The basic grid size is *D*_*s*_/8 for *x*, *y* and *z* directions. The particle is first placed at a coordinate (18D, 18D, 90D) and then suddenly released in still water, see Fig. 6a. Following the experiment by Mordant et al.^32^, the particle’s density is set as ρ_s_ = 7.85 g/cm^3^, the kinematic viscosity coefficient of the fluid is calibrated to match a particle Reynold number Re = 430 at the terminating stage.

The settling of a single particle is more suitable for verification of the terminal settling velocity, rather than performance test considering that only few immersed boundary points involved. Therefore, to validate the performance improvement by the newly proposed DIBM method, another simulation with multiple particles is carried out, which has up to 250 particles involved. The size of the computational domain is 10 cm × 100 cm × 50 cm. All the spherical particles have the same diameter of D = 1.5 cm and the same density of ρ_s_ = 1.12 g/cm^3^. The particles are uniformly placed at the top during initialization and suddenly released in still water, as shown in Fig. 6b. The particle Reynold number is estimated to be Re = 10. For both the single-particle and the multiple-particle cases, the boundary conditions at the top and bottom are set as free-slip walls and the other boundary conditions are set as no-slip walls. The mesh size is chosen to ensure that there are at least 8 grid points along the sphere’s diameter and the time step is 10^−4^ s according to the stability requirement. See more details on the configuration in Table 5.

**Table 5:** Configurations for settling simulation of a single and multiple particles.

Case	Method	Subgrid point of DIBM ($N$)	Number of IB points ($N_{{{\text{IBP}}}}$)	Number of particles	Interpolation function
Sp.1	DIBM	10	95	1	Quadratic
Mp.1	IBM	–	23,750	250	Quadratic
Mp.2	DIBM	10			

**Figure 7:**
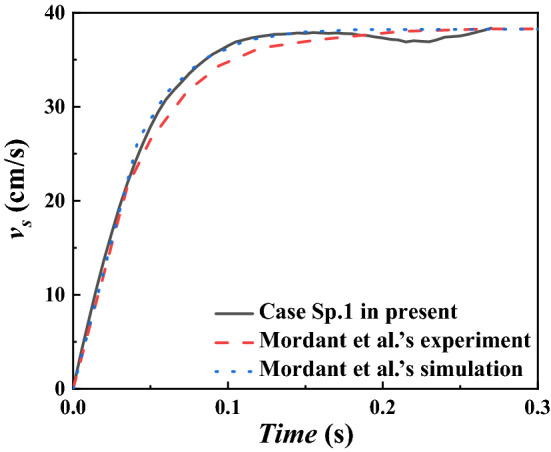
Verification of the velocity history of particle settling in still water.

The settling velocity history of a single particle is shown Fig. 7. The numerical simulation results using DIBM method agrees well with Mordant et al.^32^ in general. For the terminating settling velocity during the time at t = 0.14 s, the particle’s settling velocity is $v_{s} = 37.8{\text{cm/s}}$, which is very close to the experimental result $v_{s} = 38.3{\text{cm/s}}$. The means that the moving boundary treatment using the proposed DIBM yield simulation results with sufficient accuracy and therefore capable of handling complex problems of fluid–solid interaction, including particle-laden multiphase flows. A slight deviation is observed after t = 0.18 s in the simulation, this is because a computational domain much smaller than the experiment was adopted to save computational costs, which induces the amplified wall effects when the particle approaches the bottom. Such effect is negligible for terminal settling velocity obtained at t = 0.14 s.

**Figure 8:**
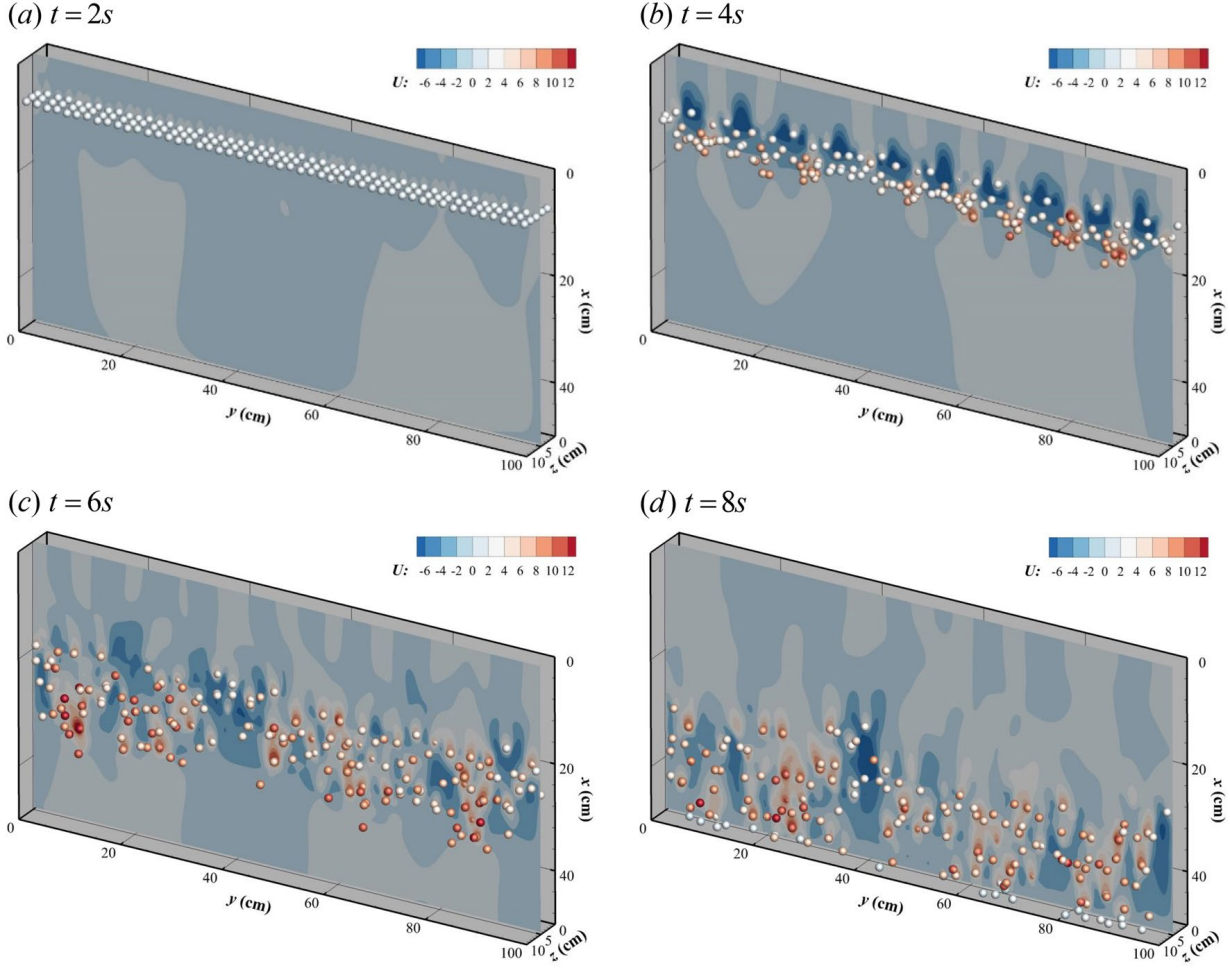
Contour of the vertical velocities during settling of multiple particles (Re = 10, unit: cm/s).

The average time consumptions for imposing the moving boundary of the 250 particles is profiled during running of the simulations, which is 2.810 s for IBM in case Mp.1 and 0.091 s for DIBM, respectively. This yields a speedup ratio of about 30. The velocity contour and particle settling process of the case Mp.2 are shown in Fig. 8. The settling process of multiple particles can be divided into 3 stages in general: uniform falling stage, transition stage, and random settling stage. During the time t = 0–2 s, it is the uniform falling stage, when all particles fall together as a whole with a roughly uniform velocity increasing with time, see Fig. 8a. The falling length of particles at this stage is about 1.5 D. During the transition stage for time around t = 2–4 s, the settling velocity of various particles begins to disperse with some falling faster and others slower, see Fig. 8b. Besides of the falling in the vertical direction, particles start moving in the horizontal directions, namely *x* and *y* directions. Such horizontal movements gradually increase the dispersion degree of the particles and finally leads into the third stage—random settling stage. At this stage, particles settle at a velocity near the terminal settling velocity at positions randomly distributed over space, see Fig. 8*c*,*d*. The settling processes and phenomena are in consistency with other similar simulations in general^20^.

### Discussions on performance and accuracy

Performance improvement for implementation of moving boundary using the immersed boundary method is meaningful and sometimes necessary. Nowadays, the computational scale of CFD simulations is growing quickly on high performance computers. New simulation contexts, like the emerging Digital Twin concepts and metaverse technology^33^, call for highly efficient real-time simulations of complex flows. Although the IBM is straightforward and easy to implement in CFD solvers, its computational cost is not negligible when thousands or even millions of moving solids, like particle-laden flows^14,20^, are involved. For CFD solver of incompressible flows using velocity–pressure decoupling technology, the pressure Poission equation usually takes most of the computational time using linear solvers like Conjugate Gradient method. However, under the framework of Cartesian structured grid, nowadays’ advanced solvers like Multigrid and FFT (fast Fourier transform) are extremely fast^34^. This further increases the proportion of IBM computation in the whole CFD solver. The proposed DIBM significantly improves the implementation performance of solid boundary treatment in CFD code by precomputing and reusing the interpolation coefficients at stenciled subgrid points, rather than repetitively calling the interpolation function every time step, which is computational expensive. In another word, the complex interpolation process is replaced by the simple looking up for prestored coefficients from arrays. The additional cost for such treatment is mainly the overhead time for memory accessing, which can be greatly decreased using the high-speed cache technique of modern CPU devices and data-parallel GPU devices.

On the other hand, the interpolation error introduced by the stenciled approximation in DIBM has been proven to be quite limited, and mostly acceptable, according to the theoretical analysis and simulation tests presented above. The error in interpolation coefficient can be further greatly decreased by increasing the subgrid resolution using more DIB points, or using higher order advanced interpolation functions. Theoretically speaking, the error can be further decreased by an additional simple interpolation among subgrid points, which of course may lead to a minor increase in computational cost. The overall error for moving solids using DIBM is usually much smaller than individual points since high frequency errors are mostly smoothed out during the process of integration over solid surface when multiple immersed boundary points are involved. Besides, as a simple and cheap way of handing moving boundaries, IBM is widely adopted in rapid prototype designs or visual effect generation in the field of computer visions, which requires more on simulation performance and implementation convenience rather than precision of results of individual points. Such simulations will particularly benefit from the proposed DIBM by an appropriate balancing between the performance and the accuracy.

Another benefit of DIBM is that it provides a generalized and modularized way for handling interpolation coefficients. Following this way, the incorporation of new interpolation functions will be more convenient because the use of the interpolation coefficients is independent from both their production (derivation), and the CFD solver. For commercial CFD solvers, DIBM provides a simple and effective interface for user development to incorporate any new interpolation functions. DIBM also facilities the sharing of various interpolation functions by different researchers via precomputed stencils for interpolation coefficients.

The methodology of the proposed DIBM highlights the fundamental idea of an alternative way of coupling between the singular forces on the IB points and the flow fields on the discretized grids. The conventional IBM can be viewed as a link between the physical quantities on the discretized background grids and the scattered IB points using continuous interpolation functions. Such functions can be of higher order to guarantee sufficient smoothness in space. However, the verification cases of DIBM demonstrate that the continuity of the interpolation functions is not always necessary from a statistical perspective. In general, the conventional IBM can be viewed as a DC system (DISCRETIZED grid + CONTINUOUS interpolation kernel), while DIBM can be viewed as a DD system (DISCRETIZED grid + DISCRETIZED interpolation kernel) by using pre-computed subgrid interpolation stencils. The idea of using more data instead of more computations is universal and the proposed framework can be referenceable for many other applications with a similar background.

## Conclusions

This study presents a high-efficiency discretized immersed boundary method (DIBM) for moving boundaries in incompressible viscous flows. The basic idea of DIBM is that by further discretizing the grid cell into finer discretized subgrids, the interpolation coefficients of the IB point can be replaced by the coefficients of the nearest subgrid points, which are precomputed and can be reused. In this way, the continuous distribution of interpolation function of IB points in grid cells could be transformed into that of finite discretized distribution. The proposed DIBM significantly improves the implementation performance of solid boundary treatment in CFD code by precomputing and reusing the interpolation coefficients at stenciled subgrid points, rather than repetitively calling the interpolation function every time step, which is computational expensive. A series of simulations are carried out to validate the accuracy and efficiency of the proposed DIBM method, including flows around a fixed and moving cylinder and settling of single and multiple particles. Simulation tests show that the speedup ratio of DIBM can reach 30–40 or even higher compared with the conventional IBM for typical moving boundary simulations like particle-laden flows, while the error is estimated to be under 1% and can be further decreased by using finer subgrid stencils. DIBM also shows higher scalability over conventional IBM when the computational scale increases.

The proposed DIBM provides is a general-purpose efficiency improving framework for boundary treatment. For simplicity, mainly the incompressible laminar flows are simulated for validations currently. However, as an alternate way to conventional IBM for computing body force terms, the method is largely independent from the Navier–stokes solver. Therefore, it’s extension to more complex turbulent flows and even Lattice Boltzmann Method (LBM) could be straightforward.

## Data Availability

The datasets used and analysed during the current study available from the corresponding author on reasonable request.
